# Detection of recurrent rearrangement breakpoints from copy number data

**DOI:** 10.1186/1471-2105-12-114

**Published:** 2011-04-21

**Authors:** Anna Ritz, Pamela L Paris, Michael M Ittmann, Colin Collins, Benjamin J Raphael

**Affiliations:** 1 Department of Computer Science, Brown University, Providence, RI, USA; 2 Department of Urology, University of California at San Francisco, San Francisco, CA, USA; 3 Department of Pathology, Baylor College of Medicine, Houston, TX, USA; 4 Vancouver Prostate Centre, Vancouver, BC, Canada; 5 Center for Computational Molecular Biology, Brown University, Providence, RI, USA

## Abstract

**Background:**

Copy number variants (CNVs), including deletions, amplifications, and other rearrangements, are common in human and cancer genomes. Copy number data from array comparative genome hybridization (aCGH) and next-generation DNA sequencing is widely used to measure copy number variants. Comparison of copy number data from multiple individuals reveals recurrent variants. Typically, the interior of a recurrent CNV is examined for genes or other loci associated with a phenotype. However, in some cases, such as gene truncations and fusion genes, the target of variant lies at the boundary of the variant.

**Results:**

We introduce Neighborhood Breakpoint Conservation (NBC), an algorithm for identifying rearrangement breakpoints that are highly conserved at the same locus in multiple individuals. NBC detects recurrent breakpoints at varying levels of resolution, including breakpoints whose location is exactly conserved and breakpoints whose location varies within a gene. NBC also identifies pairs of recurrent breakpoints such as those that result from fusion genes. We apply NBC to aCGH data from 36 primary prostate tumors and identify 12 novel rearrangements, one of which is the well-known TMPRSS2-ERG fusion gene. We also apply NBC to 227 glioblastoma tumors and predict 93 novel rearrangements which we further classify as gene truncations, germline structural variants, and fusion genes. A number of these variants involve the protein phosphatase PTPN12 suggesting that deregulation of PTPN12, via a variety of rearrangements, is common in glioblastoma.

**Conclusions:**

We demonstrate that NBC is useful for detection of recurrent breakpoints resulting from copy number variants or other structural variants, and in particular identifies recurrent breakpoints that result in gene truncations or fusion genes. Software is available at http://http.//cs.brown.edu/people/braphael/software.html.

## Background

Copy number variants (CNVs) are genomic rearrangements that result in a different number of copies of a segment of the genome, and include deletions, amplifications, and unbalanced translocations. CNVs are common in the human genome, and CNVs have been associated with several diseases [1-3]. Similarly, CNVs (also referred to as copy number aberrations, or CNAs) are found in many cancer genomes [4,5]. Thus, detection of CNVs and characterization of the gene or genes that they affect is an important task.

Array comparative genome hybridization (aCGH) [6-8] is a widely-used experimental technique for the measurement of copy number variants in genomes. aCGH involves the hybridization of differentially fluorescently labeled DNA fragments from a test genome and a reference genome to a set of genomic probes derived from the reference genome sequence. Measurements of the test:reference fluorescence ratio at each probe identify locations in the test genome that are present in lower, higher, or similar copy in the reference genome, producing a *copy number profile *of the test genome. Copy number profiles are typically compared across individuals to identify *recurrent *CNVs that are shared by multiple individuals. These recurrent CNVs may be germline polymorphisms, or in the case of cancer samples, recurrent somatic mutations. Large cohorts of aCGH data from cancer genomes (e.g. from The Cancer Genome Atlas (TCGA) [9]) provide the statistical power to identify numerous recurrent somatic CNVs. Several methods have been introduced to identify recurrent CNVs, including GISTIC [10], CoCoA [11], STAC [12], and CMDS [13]. These methods (with the exception of CMDS) first partition each copy number profile into regions (or *segments*) of equal copy number, producing a *segmentation *for each individual (see [14] for a survey of segmentation methods). Since a CNV alters the copy number of multiple adjacent probes, segmenting the copy number profile helps overcome experimental errors at each probe. These segmentations are then combined to identify aberrant intervals that are shared by multiple individuals. An implicit assumption of this approach is that the target of the CNV lies within the interval; this is the case for oncogenes that lie within amplifications or tumor suppressor genes that lie within deletions.

Some recurrent rearrangements do not target a gene within the aberrant interval, but rather target a gene or locus at the boundary of the interval. A striking example is the TMPRSS2-ERG fusion gene in prostate cancer [15]. This fusion gene results from a 3 Mb deletion on chromosome 21, where the two endpoints (or *breakpoints*) of the deletion lie in the two partner genes of the fusion. More recently, next-generation DNA sequencing has shown other fusion genes that are located at the endpoints of the CNVs (cf. figure two (b) in [16]). These and other examples motivate the development of methods that discover recurrent breakpoints rather than recurrent intervals.

We introduce a novel algorithm called Neighborhood Breakpoint Conservation (NBC) to identify recurrent breakpoints in copy number data. NBC computes the probability that a breakpoint occurs between each pair of adjacent probes over *all *possible segmentations of a single copy number profile and then combines these probabilities across multiple profiles to identify recurrent breakpoints. The probabilistic approach contrasts with the typical methods for aCGH analysis that compute only a single segmentation of a copy number profile. Consideration of a single segmentation is reasonable for identifying recurrent aberrations because large aberrations will typically overlap in different individuals as long as the segmentations reasonably approximate the true underlying copy number level. However, identification of recurrent breakpoints is more sensitive to the choice of segmentation. Due to measurement errors in individual probes, the optimal segmentation of each individual profile may not "align" across profiles. Thus it is necessary to consider multiple suboptimal segmentations. Moreover the probabilistic approach allows use to account for biological variability in the location of a breakpoint within a gene or other locus. We apply NBC to aCGH data from 36 primary prostate tumors and predict 12 CNVs, including one gene truncation and one fusion gene which is the well-known TMPRSS2-ERG fusion gene. We also apply NBC to 227 glioblastoma (GBM) tumors and predict 91 CNVs, including 23 gene truncations and 33 fusion genes. Additionally, we predict 35 germline CNVs from 107 available matched blood samples from GBM patients. A number of the somatic CNV predictions in GBM involve the protein phosphatase PTPN12, suggesting that deregulation of PTPN12 via a variety of rearrangements is common in glioblastoma. We note that NBC is readily adapted to analyze copy number profiles obtained from next-generation DNA sequencing data [17,18].

## Methods

**Figure 1:**
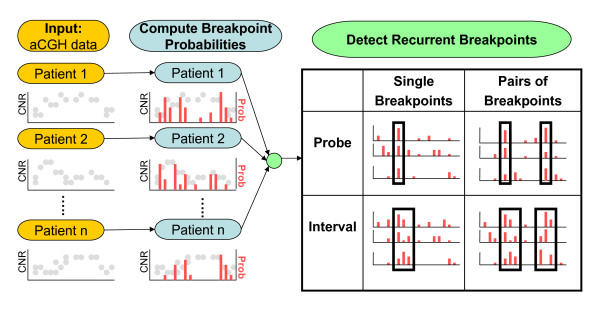
**The Neighborhood Breakpoint Conservation (NBC) algorithm**. NBC consists of two steps: computing breakpoint probabilities and recurrent breakpoint detection. Copy number ratios (CNRs) derived from aCGH data from multiple individuals are segmented using a Bayesian change-point algorithm that computes the probability of a breakpoint between adjacent probes (in red). The breakpoint probabilities are then combined to detect recurrent breakpoints (black rectangles). We identify recurrent breakpoints that occur between adjacent probes as well as recurrent breakpoints that occur within a set of probes defined by a genomic interval. To detect CNVs, we identify pairs of recurrent breakpoints.

The Neighborhood Breakpoint Conservation (NBC) algorithm takes, as input, aCGH data from many individuals and identifies recurrent breakpoints and pairs of recurrent breakpoints in a subset of the individuals (Figure 1). The first step in NBC, as in most aCGH analysis, is to segment each copy number profile into intervals of equal copy number.

While many existing methods produce a single segmentation for aCGH data [19-21], NBC uses a dynamic programming approach [22] to compute the probability  of a copy number profile **X **given a segmentation . NBC then employs a stochastic backtrace to compute the posterior probability . Using this approach, one can derive the segmentation  with maximum probability, but more importantly, one can compute the posterior probability of events of interest over *all possible segmentations *of the data. In particular, we compute the probability of a breakpoint between each pair of adjacent probes, as well as the probability of a breakpoint within a fixed interval or probes (e.g. from a gene region).

The second step of NBC is to combine breakpoint probabilities in each individual to determine breakpoints that appear in multiple individuals. Similar to [11], we use a binomial order statistic [23] to compute a *p*-value for the event that *k *or more individuals share a breakpoint between two adjacent probes. We then extend this breakpoint score to consider pairs of breakpoints that are shared by multiple individuals. Finally, we also define a score for a breakpoint that may occur anywhere within an interval of adjacent probes (e.g. a gene) that is shared by multiple individuals. We detail each of these two steps in the following sections.

### A Probability Model for Segmentation and Breakpoint Analysis

A probabilistic formulation of the segmentation problem assigns a probability to each possible segmentation of **X**. The probability of other events, such as a breakpoint occurring at a particular locus, are readily computed from this model. Probabilistic segmentation approaches have been previously applied to CNV detection [21,24-26], but we found that these methods either: require a finite number of copy number levels (as in the Bayesian Hidden Markov method of [26]); focus on probabilistic model selection rather than an explicit probabilistic model for the segmentation itself [21]; or do not perform well on high-resolution oligonucleotide arrays (see Additional File 1, Figure S3 for a comparison to [25]).

Our algorithm is based on the change-point model described in [22]. Consider a copy number profile **X **= (*X*_1_,...,*X_n_*), where *X_i _*is the log_2 _ratio of test.reference DNA at the *i*th probe. We assume that the test genome consists of an unknown number of segments *K *with corresponding copy numbers Θ = {*θ*_1_,...,*θ_K_*}. Following the usual assumptions for aCGH data [20,21,24-26], we assume that each *X_i _*is normally distributed with mean *μ_i _*and variance *σ*^2^. The variance *σ*^2 ^is a hyperparameter whose value must be set. Below we describe how we estimate this value from the data. The mean *μ_i _*equals *θ_s _*if probe *i *lies within segment *s*. Further, we assume that *X_i _*from different segments are independent. Let *l_j _*denote the number of probes in segment *j*, and let *k*_max _denote the maximum number of segments in the test genome.

We define the *breakpoint sequence ***A **= (*A*_1_, ..., *A*_*K*+1_), where *A_v _*is the index of the probe at the start of the *v *+ 1*st *segment and *A*_*K*+1 _= *n *is a "dummy" breakpoint signifying the end of the sequence (i.e. there are *K*+1 breakpoints representing *K *segments in **A**). Thus,

The unknowns in our model are the breakpoint sequence **A**, the number of segments *K*, and the segment copy numbers Θ. We assume a priori that Θ is independent of **A **and *K*. We further assume that the segment copy numbers *θ_s _*∈ Θ are independent and normally distributed with mean *μ*_0 _and variance . (The assumption that  gives a conjugate prior for  allowing us to compute some probabilities analytically. See Additional File 1, Section SA.) We assign a prior on breakpoints sequences **A **such that all **A **with *K *segments are equally likely, . Additionally, we assign a prior on the number of segments *K *such that there is a probability of  of a single segment (*K *= 1) and the remaining values of *K*, 1 <*K *≤ *k*_max_, are equally likely. Note that these priors do not make any strong assumptions about the data. essentially, the a priori assumption is that with probability  the data is produced from a single segment.

From the priors *P *(**A**|*K*) and *P *(*K *= *k*) and the values of the hyperparameter *σ*; *μ*_0_, *σ*_0_, the joint distribution *P *(**X**, **A**, Θ, *K*) can be derived (Additional File 1, Section SA).

#### Hyperparameter Estimation

The segmentation and breakpoint analysis algorithm relies on setting values for the hyperparameters *μ*_0 _(the baseline mean),  (the variance in segment copy numbers), and *σ*^2 ^(the variance in probe measurements). We describe how to estimate these from the copy number profile **X **= (*X*_1_,...,*X_n_*). First, we set *μ*_0 _to be the median of the *X_i_*. To estimate the variances  and *σ*^2^, we form sliding windows of 10 probes. Let *V *be the median of the sample variances of the windows, and let *M *be the maximum absolute difference between the sample means of the windows and *μ*_0_. We set the measurement variance *σ*^2 ^= 2*V *and the segment variance .

To test the sensitivity of our results to our particular estimates of the hyperparameters - in particular our estimates of *σ*^2 ^and  - we performed two simulations that are inspired by the simulations of [25].

**Simulation #1 **We generated an artificial chromosome with 100 probes containing a 40 probe single-copy gain (log_2 _ratio of 1) placed in the center. We then introduced various amounts of gaussian noise  in the probe measurements, setting . For each value of , we generated 100 such chromosomes.

**Simulation #2 **We generated an artificial chromosome with 100 probes with gaussian noise *N*(0, 0.5) in the probe measurements. We then introduced a 40 probe aberration at various log_2 _ratios. 0.5, 1, 2, 3, 4, 5, and 6. For each log_2 _ratio, we generated 100 such chromosomes.

A representative sample of the datasets for Simulation #1 and Simulation #2 are shown in Additional File 1, Figure S1 and S2.

We ran NBC on datasets from the two simulations with different estimates for the variances  and *σ*^2^, detailed below. To assess the quality of the resulting breakpoint predictions, we consider probe locations with Pr(breakpoint) ≥ 0.5 to be a predicted breakpoint. We assume that a predicted breakpoint detects a true breakpoint if the predicted breakpoint location is ≤ 2 probes away from the true breakpoint location. We count the number of true positive predictions (0, 1, or 2). Additionally, we count the number of false positive predictions for each dataset. We average the true positives and false positives over the 100 artificial chromosomes.

**Figure 2:**
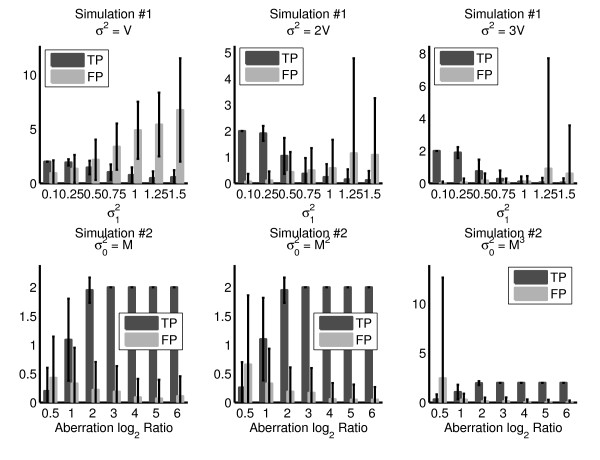
**Hyperparameter Sensitivity**. The number of true positive (TP) breakpoints (0,1, or 2) and the number of false positive (FP) breakpoints for Simulation #1 and Simulation #2 over various values of variance parameters *σ*^2 ^(top row) and  (bottom row). The bars are averaged over 100 iterations for each simulation, and error bars indicate one standard deviation.

Simulation #1 has a fixed aberration log_2 _ratio, so we set the segment variance  and we test three different values of *σ*^2^: *V*, 2*V*, and 3*V *(Figure 2 top row). Compared to our estimated value of *σ*^2 ^= 2*V*, the number of true positives is similar when *σ*^2 ^= *V *or *σ*^2 ^= 3*V *and the measurement error  is low. As  increases, setting *σ*^2 ^= *V *results in more false positives compared to our estimate *σ*^2 ^= 2*V*, while setting *σ*^2 ^= 3*V *results in fewer total predictions, including true positives. Thus, at lower measurement error the results are not particularly sensitive to the value of *σ*^2^, with our estimate *σ*^2 ^= 2*V *maintaining reasonable sensitivity and specificity and higher measurement error.

Since Simulation #2 has fixed measurement error, we set the measurement variance *σ*^2 ^= 2*V *and test three different values of , and *M*^3 ^(Figure 2 bottom row). The number of true and false positives is very similar for all three estimates of . The only exception is that when , there is a large variation in the number of false positives over the different simulated chromosomes. These simulations show that our hyperparameter estimates are reasonable, although other estimation approaches are possible.

The simulations underscore that the ability to detect the breakpoints of a segment is related to both the copy number of the segment (governed by the segment variance ) and the measurement error (governed by the variance *σ*^2^). For example, in Simulation #1 (where  is fixed), as the probe variance *σ*^2 ^increases the average number of false positive breakpoints increases while the average number of true positives remains below one. To avoid such situations, we do not segment the data and immediately report 0 breakpoints when our estimates of *σ *and *σ*_0 _satisfy *σ *≥ 3*σ*_0_.

#### Computing Breakpoint Probabilities

We compute the probability of a breakpoint between pairs of adjacent probes by sampling breakpoint sequences **A **from the distribution *P *(**A**|**X**) and counting the proportion of samples that have a breakpoint between adjacent probes. Note that the probability of a breakpoint between adjacent probes can be analytically computed (see [22]). We describe a sampling strategy, since this will generalize to the computation of the probability of breakpoints that lie within an interval or pairs or breakpoints. For notational convenience, let

*X*_[*i*:*j*] _= (*X_i_*,..., *X_j_*), *X*_(*i*:*j*] _(*X*_*i*+1_,..., *X_j_*), and *X*_[*i*:*j*) _= (*X_i_*,..., *X*_*j*-1_) The probability of **X **is(1)

where(2)

Here,  is the length (number of breakpoints) of *P *(**X**|*K *= *k*) is the probability of the data **X **given that the test genome is divided into *k *segments, and  is the probability that  consists of a single segment. The product in Equation (2) results from the segment independence assumption. The choice of a conjugate prior for *P *(*θ*) allows the integral to be analytically computed (Additional File 1, Section SA.2). However, calculating *P *(**X**|*K *= *k*) in this way requires summing over all possible breakpoint sequences **A **and is computationally infeasible. A dynamic program allows the efficient computation of this term.

#### Dynamic program

Let *P *(*X*_[*i*:*j*]_*|k*) be the probability of observing *X*_[*i*:*j*] _given that it is generated from *k *different segments. We compute this *P *(*X*_[1:*j*]_|*k*) for 1 ≤ *k *≤ *k*_max _and 1 ≤ *j *≤ *n *as follows.(3)(4)

The final row of the dynamic programming table contains *P *(**X**|*K *= *k*) for 1 ≤ *k *≤ *k*_max_, which is used in Equation (1) to compute *P *(**X**).

#### Recursive sampling

We use *P *(**X**|*K *= *k*) as well as the base case *P *(*X*_[*i*:*j*]_|1) and intermediate terms *P *(*X*_[1:*j*]_|*k*) in the dynamic program to sample exact and independent breakpoint sequences **A **using a backward sampling technique [22].

1. Draw *K *= *k *from *P *(*K *= *k*|**X**), determined by inverting *P *(**X**|*K *= *k*) using Bayes Rule.

2. Set *A*_*k*+1 _= *n*.

3. Draw *A*_*k*_, *A*_*k*-1_, ..., *A*_1 _recursively using the conditional distributions computed by the recurrences in Equation (4). Given *A_q_*, the location of the beginning of the *q*th segment, the distribution of *A*_*q*-1 _is obtained as follows.(5)

From a set of breakpoint sequences sampled in proportion to *P *(**A**|**X**), we determine the probability of a breakpoint occurring between two adjacent probes by counting the proportion of samples that contain a breakpoint at that locus. Other probabilities derived from these sampled breakpoint sequences are described in subsequent sections.

#### Runtime analysis

The base cases *P *(*X*_[*i*:*j*]_|1) require *O*(*n*^2^) computations and the dynamic program requires *O*(*nk*_max_) computations; thus computing *P *(**X**|*K *= *k*) is achieved in *O*(*n*(*n *+ *k*_max_)) time. All computations necessary to sample a breakpoint sequence **A **are already computed in the dynamic program, so sampling is linear in the number of breakpoints *K *drawn from *P *(**X**|*K *= *k*).

### Identifying Recurrent Breakpoints

After sampling breakpoint sequences for a set of individuals, we identify recurrent breakpoints that appear in many individuals at the same genomic locus. Let  be a set of copy number profiles from m individuals, where *S_j _*= (*X*_1_, ..., *X_n_*) is the copy number profile for individual *j*. We assume that the same array probes are used for each individual, i.e. the *i*th probe in individual *S_j _*is at the same location as the *i*th probe in individual *S*_*j'*_. We analyze recurrent breakpoints at two levels of resolution.

• *Recurrent probe breakpoints *occur between the same two array probes in a subset of individuals.

• *Recurrent interval breakpoints *occur within the same interval of the genome in a subset of individuals.

In addition to analyzing these types of recurrent breakpoints, we also consider pairs of recurrent breakpoints to identify recurrent CNVs. Note that these pairs may indicate *intrachromosomal *CNVs, as in the case of classic copy number aberrations like duplications and deletions, or *interchromosomal *CNVs, as in the case of (unbalanced) translocations.

#### Recurrent probe breakpoints

For each probe, we define a score that measures the presence of a breakpoint in a subset of individuals. We design this score to account for the observation that the number of breakpoints in copy-number profiles, particularly in a set of cancer samples, is highly variable. That is, in a set of cancer samples, even from the same cancer type, there will typically be highly rearranged cancer genomes with many breakpoints, and less rearranged genomes with relatively few breakpoints. This variability in the number of breakpoints is maintained following our Bayesian segmentation approach - despite the fact that we use the same flat prior for each individual - because there is strong evidence to support a larger number of breakpoints in some samples. Since there is a greater chance of recurrent breakpoints occurring randomly in a collection of highly rearranged genomes than a collection of less rearranged genomes, it is advantageous to consider the number of breakpoints in each profile when scoring recurrent breakpoints. Because the variability of number of breakpoints across different individuals is typically not well matched by a standard distribution, one approach is to use a permutation test that preserves the number and probability of breakpoints in each profile while permuting their location. We instead derive a score for recurrent probe breakpoints based on a binomial order statistic [11,23]. This score first normalizes the breakpoint probability at each probe in each individual according to the breakpoint probabilities across all probes in individual. These normalized values are then combined across multiple individuals to produce a recurrent breakpoint score.

Let *b_i _*be the event that a breakpoint lies between probes *i *and *i *+ 1; *P *(*b_i_*|*S_j_*) is the *breakpoint probability *at probe *i *in individual *S_j_*, and is computed by counting the proportion of sampled breakpoint sequences **A **that have a breakpoint between *i *and *i *+ 1. Let *ρ*_*j*_(*i*) be the fraction of probes with a higher breakpoint probability than probe *i *in individual *S_j _*(the normalized rank of probe *i*).(6)

Let *π *be a permutation of the individuals  such that . For 1 ≤ *h *≤ *m*, we wish to determine the probability that *h *or more individuals have a breakpoint at location *i*. Because of our normalization of the breakpoint probabilities in each sample, under the null hypothesis the individual scores *ρ_j_*(*i*) are independent and uniformly distributed in [0,1]. Thus, the probability that h or more individuals have a breakpoint at location *i *is given by the tail of the binomial distribution with success probability . The *p*-value for the probe location *i *is(7)

where we are only interested in scoring those breakpoints that are present in at least *h*_min _patients. Note that because the binomial order statistic is computed from the empirical distribution *ρ_j _*of breakpoint probabilities in each sample, the relative magnitude of the breakpoint probability is not used in the computation. Despite this loss of information, we found that the binomial order statistic produced reasonable results on real data (See Results below) and was more efficient than a permutation test.

Finally, we assume that a recurrent breakpoint is also conserved in the direction of the copy number change: all samples with a recurrent breakpoint are either breakpoints that go from relatively low copy number to high copy number of vice versa. A breakpoint sequence **A **defined a segmentation, and we use the mean values of each segment to determine the direction of copy number change. The copy number change is positive if the mean of the segment to the right of the breakpoint is higher than the mean of the segment to the left. We test both cases for each recurrent breakpoint, doubling the number of hypotheses we test. We control the False Discovery Rate (FDR) using the method of Benjamini and Hochberg [27].

#### Recurrent interval/gene breakpoints

We extend our approach to find recurrent breakpoints that lie within a genomic interval *W*; e.g. a gene. Unlike the recurrent probe breakpoint calculation above, where each probe was a priori equally likely to contain a breakpoint, intervals that contain more probes are a priori more likely to contain a breakpoint than intervals that contain fewer probes. To account for this, we use a log-odds score that is defined as follows. Let *b *∈ *W *be the event that one or more breakpoints lie between any pair of adjacent probes within *W*. Similarly, let *b *∉ *W *be the event that no breakpoint lies between any adjacent probes within *W*. The log-odds score ℓ_*j*_(*W*) that patient *S_j _*contains a breakpoint within *W *is(8)

The conditional probabilities *P*(*b *∈ *W *|*S_j_*) and *P*(*b *∉ = *W *|*S_j_*) describe probabilities over all possible segmentations of the copy number profile *S_j_*. *P*(*b *∈ *W *|*S_j_*) is determined by sampling breakpoint sequences **A **and counting the number of samples that contain one or more breakpoints in the interval *W*. *P*(*b *∉ = *W *|*S_j_*) is then simply 1- *P*(*b *∈ *W *|*S_j_*). The scaling factor  is computed by counting the number of ways to place breakpoints such that none of them lie in *W*:(9)(10)

Here, the last term in Equation (9) counts the number of ways to choose *k *breakpoints that do not lie in *W*. As in the recurrent breakpoint computation above, we use the binomial order statistic to combine log-odds scores across patients. First, in an analogous computation to Equation (6) we normalize the log-odds scores using the empirical cumulative distribution, which produces the normalized rank of ℓ_*j*_(W) for all *j*:(11)

Finally, using the *ρ_j_*(*W *) scores for each patient *S_j _*we compute the *p*-value *ρ*(*W *) using the binomial order statistic as in Equation (7).

For the experiments below, we define the the copy number change for an interval *W *to be positive if at least 90% of the breakpoints within the interval are positive and negative if at least 90% of the breakpoints within the interval are negative. Otherwise, we do not call a breakpoint in *W*.

#### Pairs of recurrent interval/gene breakpoints

We identify pairs of non-overlapping recurrent interval breakpoints using a log-odds score similar to Equation (8) that scores two breakpoints occurring in intervals *W*_1 _and *W*_2_. An important case we will consider is when *W*_1 _and *W*_2 _are genes. Let *b *∈ *W*_1 _be the event that a breakpoint lies between any pair of adjacent probes within *W*_1_, and Let *b' *∈ *W*_2 _be the event that a breakpoint lies between any pair of adjacent probes within *W*_2_. We define the score for intervals *W*_1 _and *W*_2 _for a particular patient *S_j_*.(12)

Each term is computed similarly to Equation (8). If *W*_1 _and *W*_2 _are on different chromosomes, the events *P *(*b *∈ *W*_1_) and *P *(*b' *∈ *W*_2_) are independent and Equations (9) and (10) are used to compute the scaling factor . If the intervals are on the same chromosome then the events are dependent, and the numerator in the scaling factor is(13)

Where

The denominator in the scaling factor is then

The *p*-value *ρ*(*W*_1_, *W*_2_) is computed by normalizing as in Equation (11) according to the empirical distribution of log-odds scores over all pairs of non-overlapping intervals and then using the binomial order statistic to determine the final *p*-value. Here, we test four hypotheses for each pair *W*_1 _and *W*_2 _by considering the four combinations of direction of copy number change: {(+, +), (-, -), (-, +), (-, -)}. Note that restricting *W*_1 _and *W*_2 _to each contain a single probe identifies pairs of recurrent probe breakpoints.

### Predicting Structural Variants, Gene Truncations, and Fusion Genes

Our statistics for single recurrent breakpoints (*ρ*(*i*) and *ρ*(*W*)) and pairs of recurrent breakpoints (*ρ*(*i*, *j*) and *ρ*(*W*_1_, *W*_2_)) provide a flexible framework to predict particular rearrangement configurations. In this paper, we classify predictions into structural variants, gene truncations, and fusion genes.

#### Structural variants

Pairs of recurrent probe breakpoints may indicate germline or somatic rearrangements that have recurrent breakpoints at the highest resolution allowed by the spacing of probes. To identify these rearrangements, we compute the pairs of recurrent probe breakpoint statistic for every pair of probes within each chromosomal arm. Note that this limits the structural variant predictions to intrachromosomal rearrangements only.

#### Gene truncations

Recurrent breakpoints found within a single gene may indicate a gene truncation, resulting in the loss of functionality for a particular gene. To predict gene truncations, we compute the recurrent interval breakpoint detection statistic, using the set of gene regions from RefSeq as our intervals of interest.

#### Fusion genes

**Figure 3:**
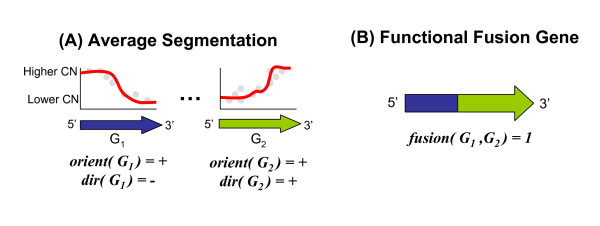
**Fusion Gene Configurations**. Fusion genes are pairs of recurrent genes that have the following configuration. (A) Each gene *G*_1 _and *G*_2 _has an associated orientation, *orient*(*G*_1_) and *orient*(*G*_2_). Additionally, each recurrent breakpoint has an associated change in relative copy number, *dir*(*G*_1_) and *dir*(*G*_2_). (B) A fusion gene joins the ends of *G*_1 _and *G*_2 _such that the 5' end of one gene is joined to the 3' end of the other gene.

Pairs of recurrent interval breakpoints found within genes suggest potential fusion genes. We compute pairs of recurrent interval breakpoints using all pairs of gene regions from RefSeq as our intervals of interest. Note that not all pairs of recurrent genes suggest functional fusion genes. For example, a rearrangement that joins the 3' end of one gene to the 3' end of another gene is typically not a functional fusion gene. Thus, we restrict our attention to pairs of interval breakpoints with particular configurations (Figure 3).

Specifically, consider a pair of recurrent intervals *G*_1 _and *G*_2 _that represent gene regions. Each gene has an orientation, *orient*(*G*_1_) ∈ {+, -} and *orient*(*G*_2_) ∈ {+, -}. Additionally, the breakpoint that lies within each recurrent interval has an associated direction of copy number change, *dir*(*G*_1_) ∈ {+, -} and *dir*(*G*_2_) ∈ {+, -}. We assume that a fusion gene contains the 5' end of one gene joined to the 3' end of the other gene and thus satisfies the following rule.(14)

### Filtering and Ranking Predictions

We apply a number of additional steps to remove and prioritize predictions. In the case of fusion genes, if there are many predictions remaining we rank these predictions by the preservation of copy number across the fusion point.

#### Removing single probe aberrations

Single probe aberrations are segments consisting of a single probe. Since these are difficult to distinguish from experimental artifacts, we remove them from further consideration. Single probe aberrations are characterized by two large changes in copy number in adjacent probes, where the segments adjacent to this aberration have a similar copy number. We identify these probes and remove them from the analysis.

#### Removing known CNVs

We remove predictions that are new known CNVs. We say that a single probe is "near" a known CNV in the Database of Genomic Variants (DGV) [28] if it is within 10 kb of a recorded copy number variant endpoint, and a gene region is "near" a known copy number variant if it is within 10 kb of a recorded copy number variant endpoint. Additionally, a pair of intrachromosomal recurrent breakpoints are near a variant if at least one of the breakpoints is within 10 kb of a recorded copy number variant endpoint and the mutual overlap between the prediction interval (defined by the pair of breakpoints) and the variant interval is greater than 50%.

#### Ranking predictions

Since fusion genes (and other recurrent pairs of breakpoints) are physically joined in the test genome, we expect the copy number of either side of the breakpoint to be the same. Thus, we rank these predictions by calculating the root mean squared difference (RMS) between the copy number levels of probes surrounding the breakpoint. Consider fusion gene predictions. we know the configuration of the gene partners, but we do not know exactly where the breakpoint lies. Thus, we determine the copy number on each side of the fusion as the average of the three flanking probes of the left gene partner and the three flanking probes of the right gene partner. If *h *patients have the breakpoint, determined by the argmax of Equation (7),  is the left-flanking copy number of the fusion and  is the right-flanking copy number of the fusion, then the RMS difference of the pair of conserved breakpoints is(15)

## Results

We applied NBC to two aCGH datasets. a collection of 36 primary prostate tumors, and 227 glioblastoma (GBM) tumors. For each dataset, we computed recurrent probe breakpoints, recurrent gene breakpoints, pairs of recurrent probe breakpoints, and pairs of recurrent gene breakpoints.

### Prostate Dataset

**Figure 4:**
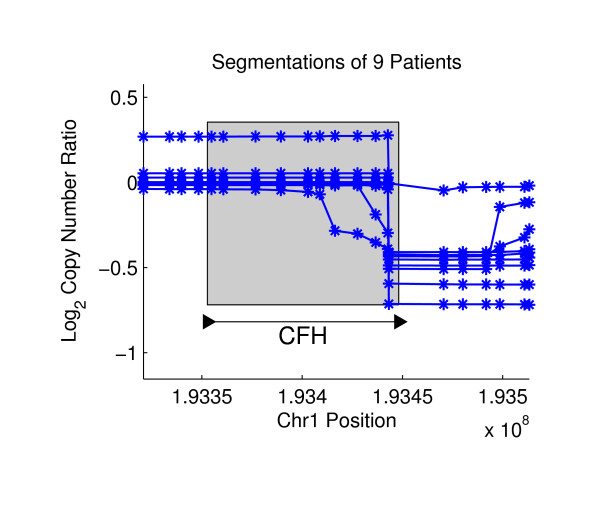
**A Predicted Gene Truncation in Prostate Cancer**. The Complement Factor H (CFH) gene on Chromosome 1 contains a recurrent gene breakpoint, suggesting the truncation of the 3' region in 9 individuals.

**Figure 5:**
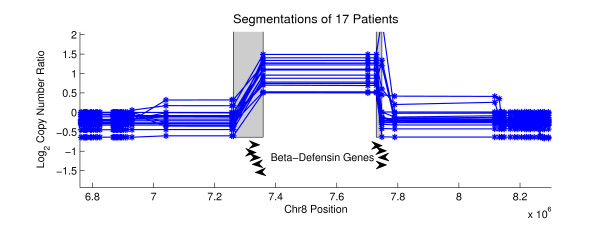
**A Predicted Rearrangement Highly Conserved at the Probe Level in Prostate Cancer**. This amplified region on Chromosome 8 lies in the DEFB locus, and the recurrent breakpoints are conserved at the probe level in 17 individuals. Arrows indicating DEFB genes are approximate.

**Figure 6:**
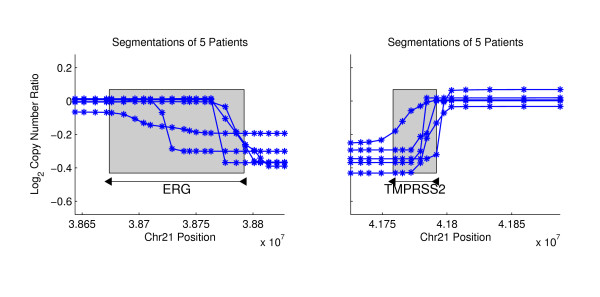
**The TMPRSS2-ERG Fusion Gene in Prostate Cancer**. We identify the TMPRSS2-ERG fusion gene in 5 prostate cancer patients. The mean segmentations for each patient (shown in blue) are computed by finding the segment parameters *θ *for each segmentation **A **drawn from the posterior distribution *P*(**A**|**X**) and then averaging these values across all segmentations.

We applied NBC to Agilent aCGH data from a collection of 36 primary prostate tumors. Each sample contained copy number ratios for 235,719 aCGH probes that were mapped to the hg17 human reference genome. We examined recurrent gene breakpoints using the gene regions from 16,162 hg17 RefSeq genes. Table 1 reports the number of predicted variants, and tables listing the breakpoint coordinates and additional information are in Additional File 2, Tables S1, S2, S3 and S4. We visualize predictions by plotting the *average *segmentation for each of the individuals that were involved of the final *p*-value computations for recurrent breakpoints in Equation (7). The average segmentation is created by averaging the segment copy numbers Θ at each probe for the sampled breakpoint sequences **A**. We predict one novel gene truncation, which occurs in the Complement factor H (CFH) gene (Figure 4). CFH encodes a protein that is secreted into the bloodstream and is essential for complement system regulation, and CFH polymorphisms are associated with macular degeneration [29]. From pairs of recurrent probes, we predict 10 novel variants, the most significant of which lies in the DEFB locus ((*p*-value = 1.3 × 10^-33^, Figure 5). DEFB genes have been associated with the risk of prostate cancer [30], and this locus lies near many known CNVs, complicating the study of nearby genes. We predict only one fusion gene, the well-known TMPRSS2-ERG fusion gene, which we detect in 5 patients with a *p*-value of 2.7 × 10^-10 ^(Figure 6). The TMPRSS2-ERG fusion gene has an RMS difference of 0.2520.

**Table 1:** Predicted Recurrent Breakpoints in 36 Prostate Samples.

Breakpoint Type	Rearrangement Type(s)	# Predicted	# in DGV	# Novel
Recurrent Probes	Highly Conserved Breakpoints	80	66	14

Recurrent Genes	Gene Truncations	6	5	1

Pairs of Recurrent Probes	Germline or Somatic Structural Variants	38	28	10

Pairs of Recurrent Genes	Intrachromosomal Fusion Genes	2	1	1

With Fusion Gene Config.*	Interchromosomal Fusion Genes	2	2	0

Breakpoint types are described by the indicated rearrangement type. '# Predicted' is the number of predictions that are significant with FDR < 0.01. '# in DGV' counts the breakpoints near known structural variants in the Database of Genomic Variants (DGV). '# Novel' is the number of predictions that are not near any known variant in DGV.

* Novel pairs of recurrent gene breakpoints consistent with the fusion gene configuration.

#### Comparison to Segmentation Approaches

To demonstrate the importance of breakpoint uncertainty in computing recurrent breakpoints, we compared our fusion gene predictions to those obtained using a single segmentation for each individual. We segmented copy number profiles from each individual using Circular Binary Segmentation (CBS) [19] (Additional File 1, Section SB). CBS returns a single segmentation (and thus a set of breakpoints) for each individual. From these sets of breakpoints, for each pair of genes from the same chromosome, we counted the number of patients with a breakpoint in each gene. Only two individuals had a pair of breakpoints within TMPRSS2 and ERG from the CBS segmentations (Additional File 1 Figure S4). Further, there are 5 fusion gene predictions that occur in two individuals after applying the filters described previously, and zero predictions that occur in more than two individuals. Since no other common fusion genes in prostate cancer are known, we assume that these remaining predictions are false positives. Thus, NBC is more sensitive and specific in fusion gene identification.

### Glioblastoma Dataset

We next applied our method to Agilent 244 K aCGH data of glioblastoma (GBM) tumors from The Cancer Genome Atlas [9]. Data was collected from 233 GBM patients, including 227 tumor samples and 107 matched blood samples. Each sample contains 227,612 aCGH probes across the hg18 human reference genome. Gene regions from 16,162 hg18 RefSeq genes were used to determine recurrent gene breakpoints. Classification of breakpoints in the tumor samples and filtering of the predictions were performed as above. Additionally, to restrict attention to somatic breakpoints we remove from consideration any recurrent breakpoints found in the tumor samples that also appear in the blood samples. When identifying recurrent probe breakpoints in the blood samples, we increase the False Discovery Rate (FDR) from 0.01 to 0.1 to more aggressively filter recurrent breakpoints in tumor samples. Table 2 reports the number of predicted variants, and tables listing the breakpoint coordinates and additional information are in Additional File 2, Tables S5, S6, S6 and S8.

**Table 2:** Predicted Recurrent Breakpoints in 227 GBM Samples and 107 Blood Samples.

Breakpoint Type	Rearrangement Type(s)	# Predicted	# in DGV	# in Blood	# Novel
Recurrent Probes in Tumor	Highly Conserved Breakpoints	538	343	13	189

Recurrent Genes in Tumor	Gene Truncations	92	69	23	23

Pairs of Recurrent Probe in Blood*	Germline Structural Variants	88	53	N/A	35

Pairs of Recurrent Genes in Tumor w/Fusion Gene Config. **	Intrachromosomal Fusion Genes	75	45	5	7
	Interchromosomal Fusion Genes	396	316	53	26

Columns are described in Table 1, except for '# in Blood' which indicates the number of predictions that also appear in the blood samples and are ignored as somatic predictions.

* FDR is increased to < 0.1 for blood samples.

** Novel pairs of recurrent gene breakpoints consistent with the fusion gene configuration.

**Figure 7:**
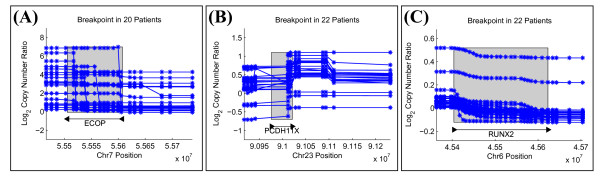
**Predicted Gene Truncations in GBM**. These three recurrent gene breakpoints found on Chromosome 7, Chromosome X, and Chromosome 6 respectively suggest truncations of genes associated with glioblastoma or other neuronal diseases. (A) The recurrent breakpoint in ECOP has a large change in copy number; this gene is near EGFR and is the breakpoint location for the EGFR amplification. (B) PCDH11X appears to arise from a short deletion within a relatively amplified region, though the deletion breakpoint varies within the PCDH11X gene region. (C) RUNX2 contains two probe locations with recurrent probe breakpoints that each have small copy number change at approximately 45.42 Mb and 45.58 Mb.

**Figure 8:**
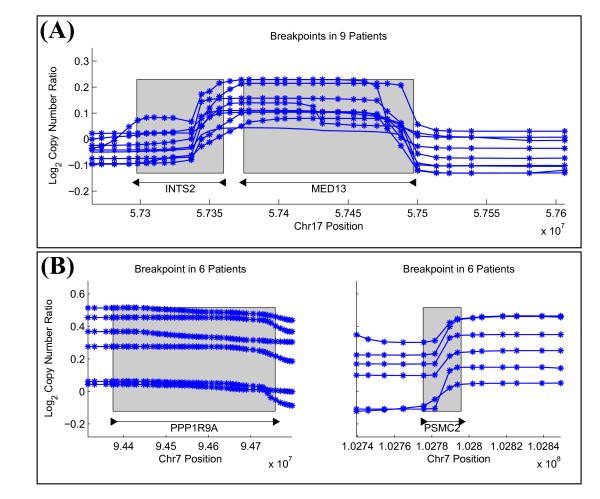
**Predicted Intrachromosomal Fusion Genes in GBM**. (A) The INTS2-MED13 rearrangement on Chromosome 17 is identified in 9 individuals and arises from an amplification. A tandem duplication that affects the 3' end of MED13 and the 5' end of INTS2 will fuse the promoter region of INTS2 to MED13. (B) The PPP1R9A-PSMC2 rearrangement on Chromosome 7 is identified in 6 individuals and arises from a deletion.

**Figure 9:**
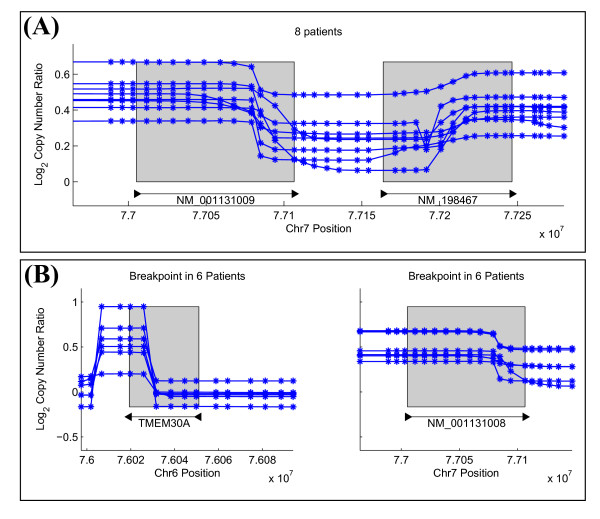
**Predicted Fusion Genes with PTPN12 as a Gene Partner**. (A) The predicted intrachromosomal fusion gene PTPN12/RSBN1L is one of two predicted intrachromosomal fusion genes. This fusion gene arises from a deletion within an amplified region, and is only present in 8 individuals out of 16 that have some rearrangement with PTPN12. (B) The predicted interchromosomal fusion gene TMEM30A-PTPN12 is one of 8 predicted interchromsomal fusion genes. While the breakpoint in TMEM30A appears to arise due to a short amplification, a translocation occurring after an amplification (where all of TMEM30A is amplified) may also explain this fusion gene signature.

We predict 23 gene truncations from the tumor samples, three of which are shown in Figure 7. Each of these has some support in the literature for an association with glioblastoma or other neuronal diseases. ECOP is co-amplified with EGFR in glioblastoma as well as other cancers [31,32], RUNX2 is expressed in glioblastoma cells [33], and PCDH11X is associated with late-onset Alzheimer's disease [34]. We also predict 33 fusion genes from the tumor samples. One of these predictions involving INTS2 and MED13 might arise due to a tandem duplication whose breakpoints are within the two genes (Figure 8a). Another prediction involves PPP1R9A, which is an imprinted gene that appears in neuronal tissues and has been shown to be expressed in other embryonic tissues [35] (Figure 8b). The phosphatase PTPN12 appears highly rearranged in 16 GBM patients, and it is a partner in a surprisingly large fraction (11/33) of the fusion gene predictions (Table 3). PTPN12 is known to dephosphorylate oncogenes c-ABL and Src; thus deregulation of PTPN12 might contribute to tumor survival [36]. While the 5' end of PTPN12 appears amplified with respect to the log_2 _copy number ratios at the 3' end, many fusion gene predictions consist of a deletion of the 3' end (i.e. Figure 9a). Additionally, some fusion gene candidates might indicate multiple rearrangements, such as a translocation occurring after an amplification that results in a fusion gene configuration (Figure 9b). Due to the large number of candidate rearrangement partners of PTPN12, it might be the deregulation of PTPN12, and not necessarily any single rearrangement, that is important for GBM.

**Table 3:** Predicted Rearrangments involving PTPN12 in GBM.

	Recurrent Gene PTPN12				
Gene		Genomic Location		# Patients	
PTPN12		chr7.77004708-77106533		16	

	Intrachromosomal Fusion Gene Predictions				
5' End Gene		3' End Gene		# Patients	RMS

PTPN12	chr7.77005287-77106533	RSBN1L	chr7.77163678-77246421	8	0.1081
PTPN12	chr7.77004708-77106533	LUC7L2	chr7.138695173-138757626	8	0.2605

	Interchromosomal Fusion Gene Predictions				
5' End Gene		3' End Gene		# Patients	RMS

TMEM30A	chr6.76019357-76051074	PTPN12	chr7.77005287-77106533	6	0.1306
RNF150	chr4.142006174-142273412	PTPN12	chr7.77005287-77106533	5	0.1409
PTPN12	chr7.77005287-77106533	MED13	chr17.57374747-57497348	9	0.1906
CLK1	chr2.201425977-201434830	PTPN12	chr7.77005287-77106533	8	0.3168
ZRANB2	chr1.71301561-71319266	PTPN12	chr7.77005287-77106533	9	0.3250
PTPN12	chr7.77005287-77106533	UBR1	chr15.41022389-41185512	9	0.3475
PTPN12	chr7.77005287-77106533	LINGO1	chr15.75692423-75711712	8	0.3787
PPIL3	chr2.201443923-201460583	PTPN12	chr7.77004708-77106533	6	0.4741

The phosphatase PTPN12 appears in 10 predicted fusion genes, and is also a predicted gene truncation for 16 patients. The predictions are ranked according to the root mean squared difference (RMS) of the copy number on either side of the fusion point.

## Discussion

NBC successfully identifies known fusion genes and structural variants. For fusion genes, NBC's consideration of uncertainty and variability in the locations of breakpoints provides an advantage over methods that compare individual segmentations of copy number profiles. This advantage is mitigated for variants with highly conserved breakpoints such as germline structural variants that are common in a population. However, it is possible that NBC would be helpful for complex, or overlapping, structural variants, where recurrent breakpoints might be a stronger signal than recurrent aberrant intervals.

NBC relies on a Bayesian change point algorithm, which requires specifying both prior distributions and a few hyperparameters. The weak priors that we use do not make strong assumptions about the data. However, hyperparameter estimation for Bayesian change point algorithms remains a difficult problem, and is sensitive to the particular type of data to be segmented. While our method chooses the hyperparameters systematically from the data rather than requiring user-defined input, poor parameter estimation leads to excessive breakpoint calling if there are no breakpoints to find or if the experimental error cannot be modeled by a constant *σ*^2^. We presented one approach to estimate hyperparameters from aCGH data, but more sophisticated methods (e.g. empirical Bayesian approaches) could be used [37].

In this paper, we focused on applications of NBC to aCGH data. But NBC is equally applicable to copy number profiles generated by mapping DNA sequence reads to a reference genome [17,18]. With next generation sequencing technologies, breakpoint resolution can be much higher than most current aCGH methods, but the problems of breakpoint variability and uncertainty remain.

## Conclusions

We have introduced Neighborhood Breakpoint Conservation (NBC), an algorithm that identifies recurrent breakpoints in data from multiple individuals. NBC correctly identifies a known fusion gene (TMPRSS2-ERG) in aCGH data from 36 prostate tumors and predicts gene truncations, structural variants, and fusion genes in aCGH data from glioblastoma. We expect that application of our method to additional samples will allow us to uncover and categorize other recurrent germline and somatic rearrangements.

## Authors' contributions

PLP, MMI, and CC provided aCGH data from prostate cancer samples. AR implemented the algorithm and performed experiments. BJR conceived of the project and supervised the work. AR and BJR wrote the manuscript. All authors read and approved the manuscript.

## Supplementary Material

Additional File 1 **The Appendix includes full derivations of the segmentation model, comparisons to other segmentation algorithms, and data aquisition and implementation details**. Click here for file

Additional File 2 **Tables of all the breakpoints and pairs of breakpoints predicted for the prostate dataset and the GBM dataset**. Note that the values reported for the prostate dataset (e.g. the RMS difference) are log base 10, while the values reported for the GBM dataset are log base 2. Click here for file

## References

[B1] Pinto D Functional impact of global rare copy number variation in autism spectrum disorders Nature 2010 466 368 372 10.1038/nature09146 20531469 PMC3021798

[B2] St Clair D Copy number variation and schizophrenia Schizophr Bull 2009 35 9 12 10.1093/schbul/sbn147 18990708 PMC2643970

[B3] Choy KW Setlur SR Lee C Lau TK The impact of human copy number variation on a new era of genetic testing BJOG 2010 117 391 398 10.1111/j.1471-0528.2009.02470.x 20105165

[B4] Pinkel D Albertson DG Array comparative genomic hybridization and its applications in cancer Nat Genet 2005 37 Suppl S11 7 15920524 10.1038/ng1569

[B5] Paris PL Andaya A Fridlyand J Jain AN Weinberg V Kowbel D Brebner JH Simko J Watson JE Volik S Albertson DG Pinkel D Alers JC van der Kwast TH Vissers KJ Schroder FH Wildhagen MF Febbo PG Chinnaiyan AM Pienta KJ Carroll PR Rubin MA Collins C van Dekken H Whole genome scanning identifies genotypes associated with recurrence and metastasis in prostate tumors Hum Mol Genet 2004 13 1303 1313 10.1093/hmg/ddh155 15138198

[B6] Pinkel D Segraves R Sudar D Clark S Poole I Kowbel D Collins C Kuo WL Chen C Zhai Y Dairkee SH Ljung BM Gray JW Albertson DG High resolution analysis of DNA copy number variation using comparative genomic hybridization to microarrays Nat Genet 1998 20 2 207 11 10.1038/2524 9771718

[B7] Lucito R Healy J Alexander J Reiner A Esposito D Chi M Rodgers L Brady A Sebat J Troge J West JA Rostan S Nguyen KC Powers S Ye KQ Olshen A Venkatraman E Norton L Wigler M Representational oligonucleotide microarray analysis: a high-resolution method to detect genome copy number variation Genome Res 2003 13 10 2291 305 10.1101/gr.1349003 12975311 PMC403708

[B8] Barrett MT Scheffer A Ben-Dor A Sampas N Lipson D Kincaid R Tsang P Curry B Baird K Meltzer PS Yakhini Z Bruhn L Laderman S Comparative genomic hybridization using oligonucleotide microarrays and total genomic DNA Proc Natl Acad Sci USA 2004 101 51 17765 70 10.1073/pnas.0407979101 15591353 PMC535426

[B9] McLendon R Comprehensive genomic characterization defines human glioblastoma genes and core pathways Nature 2008 455 1061 1068 10.1038/nature07385 18772890 PMC2671642

[B10] Beroukhim R Getz G Nghiemphu L Barretina J Hsueh T Linhart D Vivanco I Lee JC Huang JH Alexander S Du J Kau T Thomas RK Shah K Soto H Perner S Prensner J Debiasi RM Demichelis F Hatton C Rubin MA Garraway LA Nelson SF Liau L Mischel PS Cloughesy TF Meyerson M Golub TA Lander ES Mellinghoff IK Sellers WR Assessing the significance of chromosomal aberrations in cancer: methodology and application to glioma Proc Natl Acad Sci USA 2007 104 20007 20012 10.1073/pnas.0710052104 18077431 PMC2148413

[B11] Ben-Dor A Lipson D Tsalenko A Reimers M Baumbusch LO Barrett MT Weinstein JN Børresen-Dale AL Yakhini Z Framework for Identifying Common Aberrations in DNA Copy Number Data RECOMB 2007 2007 LNBI 4453 122 136

[B12] Diskin SJ Eck T Greshock J Mosse YP Naylor T Stoeckert CJ Weber BL Maris JM Grant GR STAC: A method for testing the significance of DNA copy number aberrations across multiple array-CGH experiments Genome Res 2006 16 1149 1158 10.1101/gr.5076506 16899652 PMC1557772

[B13] Zhang Q Ding L Larson DE Koboldt DC McLellan MD Chen K Shi X Kraja A Mardis ER Wilson RK Borecki IB Province MA CMDS: a population-based method for identifying recurrent DNA copy number aberrations in cancer from high-resolution data Bioinformatics 2010 26 464 469 10.1093/bioinformatics/btp708 20031968 PMC2852218

[B14] Lai WR Johnson MD Kucherlapati R Park PJ Comparative analysis of algorithms for identifying amplifications and deletions in array CGH data Bioinformatics 2005 21 19 3763 70 10.1093/bioinformatics/bti611 16081473 PMC2819184

[B15] Tomlins SA Rhodes DR Perner S Dhanasekaran SM Mehra R Sun XW Varambally S Cao X Tchinda J Kuefer R Lee C Montie JE Shah RB Pienta KJ Rubin MA Chinnaiyan AM Recurrent fusion of TMPRSS2 and ETS transcription factor genes in prostate cancer Science 2005 310 5748 644 8 10.1126/science.1117679 16254181

[B16] Campbell PJ Stephens PJ Pleasance ED O'Meara S Li H Santarius T Stebbings LA Leroy C Edkins S Hardy C Teague JW Menzies A Goodhead I Turner DJ Clee CM Quail MA Cox A Brown C Durbin R Hurles ME Edwards PA Bignell GR Stratton MR Futreal PA Identification of somatically acquired rearrangements in cancer using genome-wide massively parallel paired-end sequencing Nat Genet 2008 40 722 729 10.1038/ng.128 18438408 PMC2705838

[B17] Chiang DY Getz G Jaffe DB O'Kelly MJ Zhao X Carter SL Russ C Nusbaum C Meyerson M Lander ES High-resolution mapping of copy-number alterations with massively parallel sequencing Nat Methods 2009 6 99 103 10.1038/nmeth.1276 19043412 PMC2630795

[B18] Yoon S Xuan Z Makarov V Ye K Sebat J Sensitive and accurate detection of copy number variants using read depth of coverage Genome Res 2009 19 1586 1592 10.1101/gr.092981.109 19657104 PMC2752127

[B19] Olshen AB Venkatraman ES Lucito R Wigler M Circular binary segmentation for the analysis of array-based DNA copy number data Biostatistics 2004 5 4 557 572 10.1093/biostatistics/kxh008 15475419

[B20] Picard F Robin S Lavielle M Vaisse C Daudin JJ A statistical approach for array CGH data analysis BMC Bioinformatics 2005 6 27 10.1186/1471-2105-6-27 15705208 PMC549559

[B21] Zhang NR Siegmund DO A modified Bayes information criterion with applications to the analysis of comparative genomic hybridization data Biometrics 2007 63 22 32 10.1111/j.1541-0420.2006.00662.x 17447926

[B22] Liu JS Lawrence CE Bayesian inference on biopolymer models Bioinformatics 1999 15 38 52 10.1093/bioinformatics/15.1.38 10068691

[B23] David H Nagaraja H Hoboken NJ Order Statistics 2003 3 John Wiley

[B24] Barry D Hartigan JA A Bayesian Analysis for Change Point Problems Journal of the American Statistical Association 1993 88 421 309 319 10.2307/2290726

[B25] Erdman C Emerson JW A fast Bayesian change point analysis for the segmentation of microarray data Bioinformatics 2008 24 2143 2148 10.1093/bioinformatics/btn404 18667443

[B26] Guha S Li Y Neuberg D Bayesian Hidden Markov Modeling of Array CGH Data 2008 103 485 497 10.1198/016214507000000923 PMC3286622 22375091

[B27] Benjamini Y Hochberg Y Controlling the False Discovery Rate: A Practical and Powerful Approach to Multiple Testing Journal of the Royal Statistical Society. Series B (Methodological) 1995 57 289 300

[B28] Iafrate AJ Feuk L Rivera MN Listewnik ML Donahoe PK Qi Y Scherer SW Lee C Detection of large-scale variation in the human genome Nat Genet 2004 36 949 951 10.1038/ng1416 15286789

[B29] Christen WG Glynn RJ Chew EY Albert CM Manson JE Folic acid, pyridoxine, and cyanocobalamin combination treatment and age-related macular degeneration in women: the Women's Antioxidant and Folic Acid Cardiovascular Study Arch Intern Med 2009 169 335 341 10.1001/archinternmed.2008.574 19237716 PMC2648137

[B30] Huse K Taudien S Groth M Rosenstiel P Szafranski K Hiller M Hampe J Junker K Schubert J Schreiber S Birkenmeier G Krawczak M Platzer M Genetic variants of the copy number polymorphic beta-defensin locus are associated with sporadic prostate cancer Tumour Biol 2008 29 83 92 10.1159/000135688 18515986

[B31] Eley GD Reiter JL Pandita A Park S Jenkins RB Maihle NJ James CD A chromosomal region 7p11.2 transcript map: its development and application to the study of EGFR amplicons in glioblastoma Neurooncology 2002 4 86 94 10.1093/neuonc/4.2.86 PMC1920657 11916499

[B32] Baras A Yu Y Filtz M Kim B Moskaluk CA Combined genomic and gene expression microarray profiling identifies ECOP as an upregulated gene in squamous cell carcinomas independent of DNA amplification Oncogene 2009 28 2919 2924 10.1038/onc.2009.150 19525979

[B33] Vladimirova V Waha A Luckerath K Pesheva P Probstmeier R Runx2 is expressed in human glioma cells and mediates the expression of galectin-3 J Neurosci Res 2008 86 2450 2461 10.1002/jnr.21686 18438928

[B34] Carrasquillo MM Zou F Pankratz VS Wilcox SL Ma L Walker LP Younkin SG Younkin CS Younkin LH Bisceglio GD Ertekin-Taner N Crook JE Dickson DW Petersen RC Graff-Radford NR Younkin SG Genetic variation in PCDH11X is associated with susceptibility to late-onset Alzheimer's disease Nat Genet 2009 41 192 198 10.1038/ng.305 19136949 PMC2873177

[B35] Nakabayashi K Makino S Minagawa S Smith AC Bamforth JS Stanier P Preece M Parker-Katiraee L Paton T Oshimura M Mill P Yoshikawa Y Hui CC Monk D Moore GE Scherer SW Genomic imprinting of PPP1R9A encoding neurabin I in skeletal muscle and extra-embryonic tissues J Med Genet 2004 41 601 608 10.1136/jmg.2003.014142 15286155 PMC1735868

[B36] Meng F Henson R Lang M Wehbe H Maheshwari S Mendell JT Jiang J Schmittgen TD Patel T Involvement of human micro-RNA in growth and response to chemotherapy in human cholangiocarcinoma cell lines Gastroenterology 2006 130 2113 2129 10.1053/j.gastro.2006.02.057 16762633

[B37] Lian H Thompson WA Thurman R Stamatoyannopoulos JA Noble WS Lawrence CE Automated mapping of large-scale chromatin structure in ENCODE Bioinformatics 2008 24 1911 1916 10.1093/bioinformatics/btn335 18591192 PMC2519158

