# Supplementary material for: Detection of recurrent rearrangement breakpoints from copy number data
Source: BMC Bioinformatics. 2011 Apr 21;12:114. doi: 10.1186/1471-2105-12-114 (PMC3112242; doi:10.1186/1471-2105-12-114)
Supplement: Additional File 1 — The Appendix includes full derivations of the segmentation model, comparisons to other segmentation algorithms, and data aquisition and implementation details. [file 1471-2105-12-114-S1.PDF]

Appendix for

Detection of Recurrent Rearrangement Breakpoints from Copy Number Data

Anna Ritz[1*], Pamela L. Paris[2], Michael M. Ittmann[3], Colin Collins[4], and Benjamin J. Raphael[1,5*]

[1]Department of Computer Science, Brown University, Providence, RI, USA.

[2]Department of Urology, University of California at San Francisco, San Francisco, CA, USA.

[3]Department of Pathology, Baylor College of Medicine, Houston, TX, USA.

[4]Vancouver Prostate Centre, Vancouver, BC, Canada.

[5]Center for Computational Molecular Biology, Brown University, Providence RI, USA.

[*]Corresponding authors {aritz@cs.brown.edu, braphael@brown.edu}.

Contents

A Derivations **1**

A.1 Priors 1

A.2 Derivation of $P(X_{[A_{v-1}:A_v)}|\mu_0, \sigma_0^2, \sigma^2, K = 1)$ 1

A.3 Hyperparameter Estimation 3

B Algorithm Comparisons **3**

B.1 Comparison to BCP 3

B.2 Comparison to CBS 8

C Implementation Details **8**

D Data Access **8**

A Derivations

We present the full derivations for the Bayesian change-point algorithm and the complete statistics for computing recurrent breakpoints. The segmentation model is based on [5], using a normal distribution instead of a Dirichlet distribution. The recurrent breakpoint scores are similar to the Context-Corrected Penetrance of [2].

A.1 Priors

We assume that a priori all segmentations with K segments are equally likely, and have prior probability inversely proportional to the number of ways to segment the sequence into K parts,

$$P(\mathbf{A}|K) = \binom{n}{K}^{-1}. \quad (1)$$

We also assign a prior probability to the number of segments,

$$P(K) = \begin{cases} 1/2 & K = 1 \\ 1/(2(k_{\max})) & 1 < K \le k_{\max} \end{cases} \quad (2)$$

A.2 Derivation of $P(X_{[A_{v-1}:A_v)}|\mu_0, \sigma_0^2, \sigma^2, K = 1)$

$P(X_{[A_{v-1}:A_v)}|\mu_0, \sigma_0^2, \sigma^2, K = 1)$ can be computed analytically. For simplicity, we show the derivation for $P(\mathbf{X}|\mu_0, \sigma_0^2, \sigma^2, K = 1)$ rather than $X_{[A_{v-1}:A_v)}$, where $|\mathbf{X}| = n$. The following identity will be useful:

$$\sum_{i=1}^{n}(X_i-\mu)^2=\sum_{i=1}^{n}(X_i-\overline{\mathbf{X}})^2+n(\overline{\mathbf{X}}-\mu)^2. \tag{3}$$

Following Theorem 9.6 in [7] (also mentioned on p. 97 in [3]),

$$P(\mu|\mathbf{X})=N(\mu^*,(\sigma^*)^2), \text{ where} \tag{4}$$

$$\mu^*=\frac{n\overline{\mathbf{X}}\sigma_0^2+\mu_0\sigma^2}{n\sigma_0^2+\sigma^2}, \tag{5}$$

$$\sigma^*=\sqrt{\frac{\sigma_0^2\sigma^2}{n\sigma_0^2+\sigma^2}}, \text{ and} \tag{6}$$

$$\overline{\mathbf{X}}=\frac{1}{n}\sum_{i=1}^{n}X_i. \tag{7}$$

Each observation X_i is normally distributed with mean μ_i and the prior $P(\mu)$ is also normally distributed. We can multiply the $n+1$ normals and produce

$$P(\mathbf{X}|\mu)P(\mu)=\frac{1}{(2\pi)^{(n+1)/2}\sigma^n\sigma_0}\times\exp\left[\frac{-1}{2\sigma^2}\sum_{i=1}^{n}(X_i-\overline{\mathbf{X}})^2\right] \tag{8}$$

$$\times\exp\left[\frac{-1}{2}\left(\frac{n(\overline{\mathbf{X}}-\mu)^2}{\sigma^2}+\frac{(\mu-\mu_0)^2}{\sigma_0^2}\right)\right]. \tag{9}$$

Expanding the exponent in (9) results in

$$\frac{-1}{2}\left[\left(\frac{n(\overline{\mathbf{X}}-\mu)^2}{\sigma^2}+\frac{(\mu-\mu_0)^2}{\sigma_0^2}\right)\right] \tag{10}$$

$$=\frac{-1}{2}\left[\mu^2\left(\frac{n\sigma_0^2+\sigma^2}{2\sigma^2\sigma_0^2}\right)-2\mu\left(\frac{n\sigma_0^2\overline{\mathbf{X}}+\mu_0\sigma^2}{\sigma^2\sigma_0^2}\right)+\left(\frac{n\sigma_0^2\overline{\mathbf{X}}^2+\mu_0^2\sigma^2}{\sigma^2\sigma_0^2}\right)\right] \tag{11}$$

$$=\frac{-1}{2}\left[\mu^2\left(\frac{1}{(\sigma^*)^2}\right)-2\mu\left(\frac{n\sigma_0^2\overline{\mathbf{X}}+\mu_0\sigma^2}{\sigma^2\sigma_0^2}\right)+\left(\frac{n\sigma_0^2\overline{\mathbf{X}}^2+\mu_0^2\sigma^2}{\sigma^2\sigma_0^2}\right)\right] \tag{12}$$

$$=\frac{-1}{2(\sigma^*)^2}\left[\mu^2-2\mu\left(\frac{n\sigma_0^2\overline{\mathbf{X}}+\mu_0\sigma^2}{n\sigma_0^2+\sigma^2}\right)+\left(\frac{n\sigma_0^2\overline{\mathbf{X}}^2+\mu_0^2\sigma^2}{n\sigma_0^2+\sigma^2}\right)\right] \tag{13}$$

$$=\frac{-1}{2(\sigma^*)^2}\left[\mu^2-2\mu\mu^*+(\mu^*)^2-\left(\frac{n\sigma_0^2\sigma_0^2(\mu_0-\overline{\mathbf{X}})^2}{(n\sigma_0^2+\sigma^2)^2}\right)\right] \tag{14}$$

$$=\frac{-1}{2(\sigma^*)^2}\left[\mu^2-2\mu\mu^*+(\mu^*)^2-\left(\frac{(\sigma^*)^2n(\mu_0-\overline{\mathbf{X}})^2}{n\sigma_0^2+\sigma^2}\right)\right] \tag{15}$$

$$=\frac{-1}{2(\sigma^*)^2}\left[\mu^2-2\mu\mu^*+(\mu^*)^2\right]+\left(\frac{n(\mu_0-\overline{\mathbf{X}})^2}{2(n\sigma_0^2+\sigma^2)}\right). \tag{16}$$

$$\tag{17}$$

Plugging this into $P(\mathbf{X}|\mu)P(\mu)$ yields

$$P(\mathbf{X}|\mu)P(\mu)=\frac{\exp\left[-\frac{\sum_{i=1}^{n}(X_i-\overline{\mathbf{X}})^2}{2\sigma^2}+\frac{n(\mu_0-\overline{\mathbf{X}})^2}{2(n\sigma_0^2+\sigma^2)}\right]}{(2\pi)^{(n+1)/2}\sigma^n\sigma_0}\times\exp\left[\frac{-(\mu-\mu^*)^2}{2(\sigma^*)^2}\right]. \tag{18}$$

Marginalizing over μ finally gives the probability of the data $\mathbf{X}$ given that it is a single segment; we need to multiply the top and bottom by $\sqrt{2\pi(\sigma^*)^2}$ to accomplish this.

$$P(\mathbf{X}|K=1) = \frac{\sqrt{2\pi(\sigma^*)^2}\exp\left[-\frac{\sum_{i=1}^n (X_i-\overline{\mathbf{X}})^2}{2\sigma^2} + \frac{n(\mu_0-\overline{\mathbf{X}})^2}{2(n\sigma_0^2+\sigma^2)}\right]}{(2\pi)^{(n+1)/2}\sigma^n\sigma_0} \tag{19}$$

$$\times \int \frac{1}{\sqrt{2\pi(\sigma^*)^2}}\exp\left[\frac{-(\mu-\mu^*)^2}{2(\sigma^*)^2}\right] \tag{20}$$

$$= \frac{\sqrt{2\pi(\sigma^*)^2}\exp\left[-\frac{\sum_{i=1}^n (X_i-\overline{\mathbf{X}})^2}{2\sigma^2} + \frac{n(\mu_0-\overline{\mathbf{X}})^2}{2(n\sigma_0^2+\sigma^2)}\right]}{(2\pi)^{(n+1)/2}\sigma^n\sigma_0} \tag{21}$$

$$= \sqrt{\frac{\sigma^2\sigma_0^2}{(n\sigma_0^2+\sigma^2)}}\frac{\exp\left[-\frac{\sum_{i=1}^n (X_i-\overline{\mathbf{X}})^2}{2\sigma^2} + \frac{n(\mu_0-\overline{\mathbf{X}})^2}{2(n\sigma_0^2+\sigma^2)}\right]}{(2\pi\sigma^2)^{n/2}\sigma_0} \tag{22}$$

$$= \underbrace{\sqrt{\frac{\sigma^2}{(n\sigma_0^2+\sigma^2)}}}_{\text{noise-to-signal ratio } \sqrt{w}} \quad \frac{\exp\left[-\frac{\sum_{i=1}^n (X_i-\overline{\mathbf{X}})^2}{2\sigma^2} + \frac{n(\mu_0-\overline{\mathbf{X}})^2}{2(n\sigma_0^2+\sigma^2)}\right]}{(2\pi\sigma^2)^{n/2}} \tag{23}$$

Define the noise-to-signal ratio $w(l_v) = \sigma^2/(n_v\sigma_0^2+\sigma^2)$, which depends on the length l_v of the segment. We can now write down the probability that $\mathbf{X}$ is generated from $k>1$ segments:

$$P(\mathbf{X}) = \sum_{k=1}^{k_{\max}} P(\mathbf{X}|K=k)P(K=k)$$

$$P(\mathbf{X}|K=k) = \sum_{\mathbf{A}:||\mathbf{A}||=k}\left[\int P(\mathbf{X},\mathbf{A}|\Theta)P(\Theta)d\Theta\right]$$

$$= \sum_{\mathbf{A}:||\mathbf{A}||=k}\left[\prod_{v=1}^{k} P(\mathbf{X}_{[A_{v-1}:A_v)}|\mu_0,\sigma_0^2,\sigma^2,K=1)\right] \tag{24}$$

$$\propto \sum_{\mathbf{A}:||\mathbf{A}||=k}\left[\prod_{v=1}^{k}\left[\sqrt{w(l_v)}\exp\left[\frac{\sum_{i=A_{k-1}}^{A_k-1}(X_i-\overline{X}_{[A_{k-1}:A_k)})^2 + w(l_v)l_v(\mu_0-\overline{X}_{[A_{k-1}:A_k)})^2}{2\sigma^2}\right]\right]\right]. \tag{25}$$

A.3 Hyperparameter Estimation

To test the sensitivity of our choice of hyperparameters, particularly the variances σ_0^2 and σ^2, we performed two simulations similar to the simulations of [4].

Simulation #1 We generated an artificial chromosome of length 100 with a 40 probe single-copy gain ($\log_2$ ratio of 1) placed in the center. We then introduced various amounts of gaussian noise $N(0,\sigma_1^2)$ in the probe measurements, where $\sigma_1^2 = 0.1, 0.25, 0.5, 1, 1.25$, or 1.5. For each value of σ_1^2, we generated 100 such chromosomes.

Simulation #2 We generated an artificial chromosome of length 100 with gaussian noise $N(0,0.5)$ in the probe measurements. We then introduced a 40 probe aberration at various $\log_2$ ratios: $0.5, 1, 2, 3, 4, 5$, and 6. For each $\log_2$ ratio, we generated 100 such chromosomes.

A representative sample of the datasets for Simulation #1 is shown in Figure 1 and for Simulation #2 is shown in Figure 2.

B Algorithm Comparisons

B.1 Comparison to BCP

The method by Erdman and Emerson, BCP [4], applies the change-point algorithm by Barry and Hartigan [1] to aCGH data using a different generative model than the segmentation portion of NBC. Their segmentations tend to have many more probes with high breakpoint probabilities than expected, even on relatively smooth data. Figure 3 shows the mean segmentations and breakpoint probabilities averaged over the 5 patients that contain the TMPRSS-ERG fusion gene in Prostate cancer. While the fusion gene is clearly visible from the segmentations from BCP, the fusion gene cannot be clearly identified from the breakpoint probabilities of BCP.

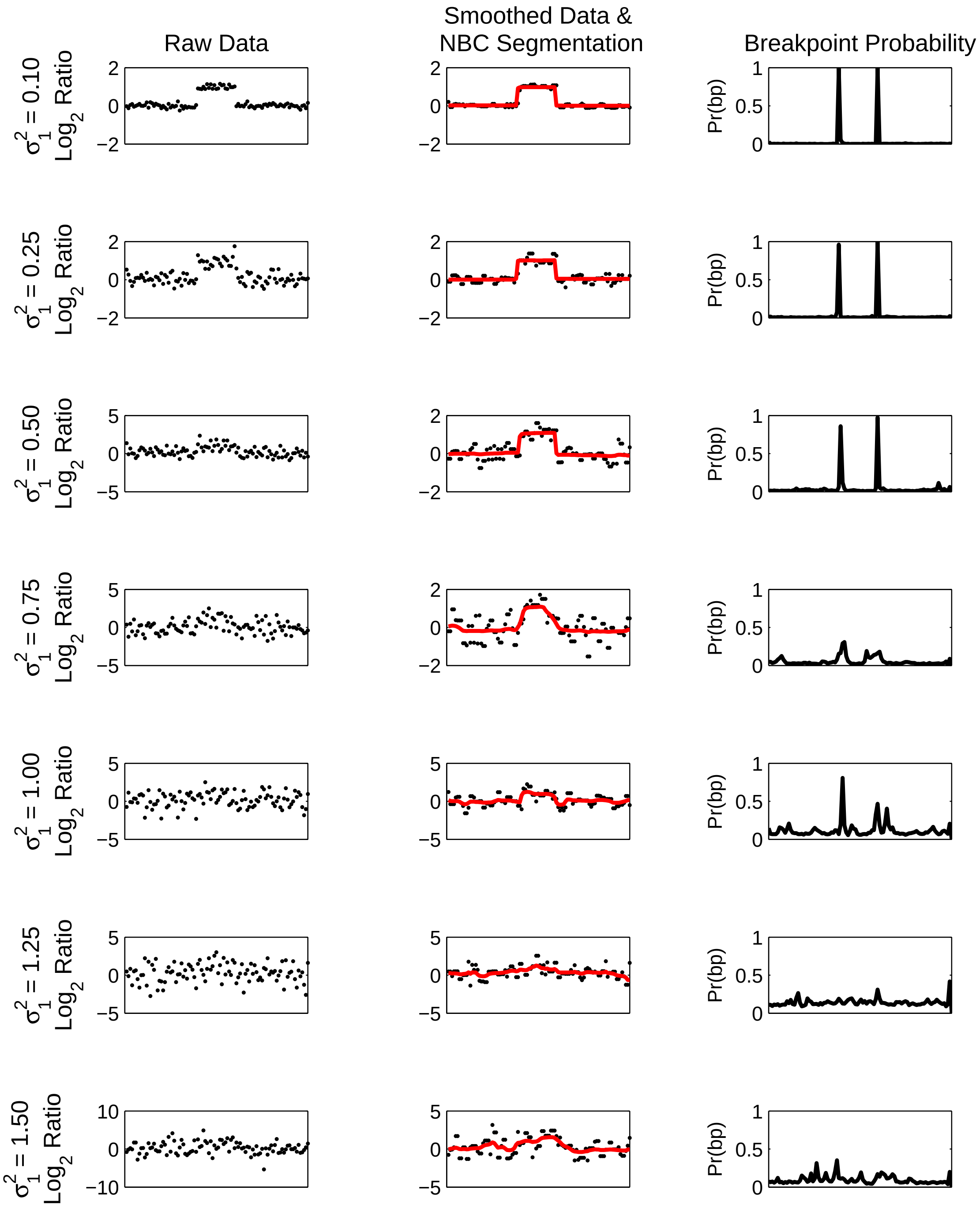

Figure 1: A single artificial chromosome from Simulation #1 with gaussian noise $N(0, \sigma_1^2)$ for $\sigma_1^2 = 0.1, 0.25, 0.5, 1, 1.25$ or 1.5. The first column shows the raw data for a particular σ_1^2. The second column shows the smoothed data and the average NBC segmentation (red). The third column shows the breakpoint probability at each location.

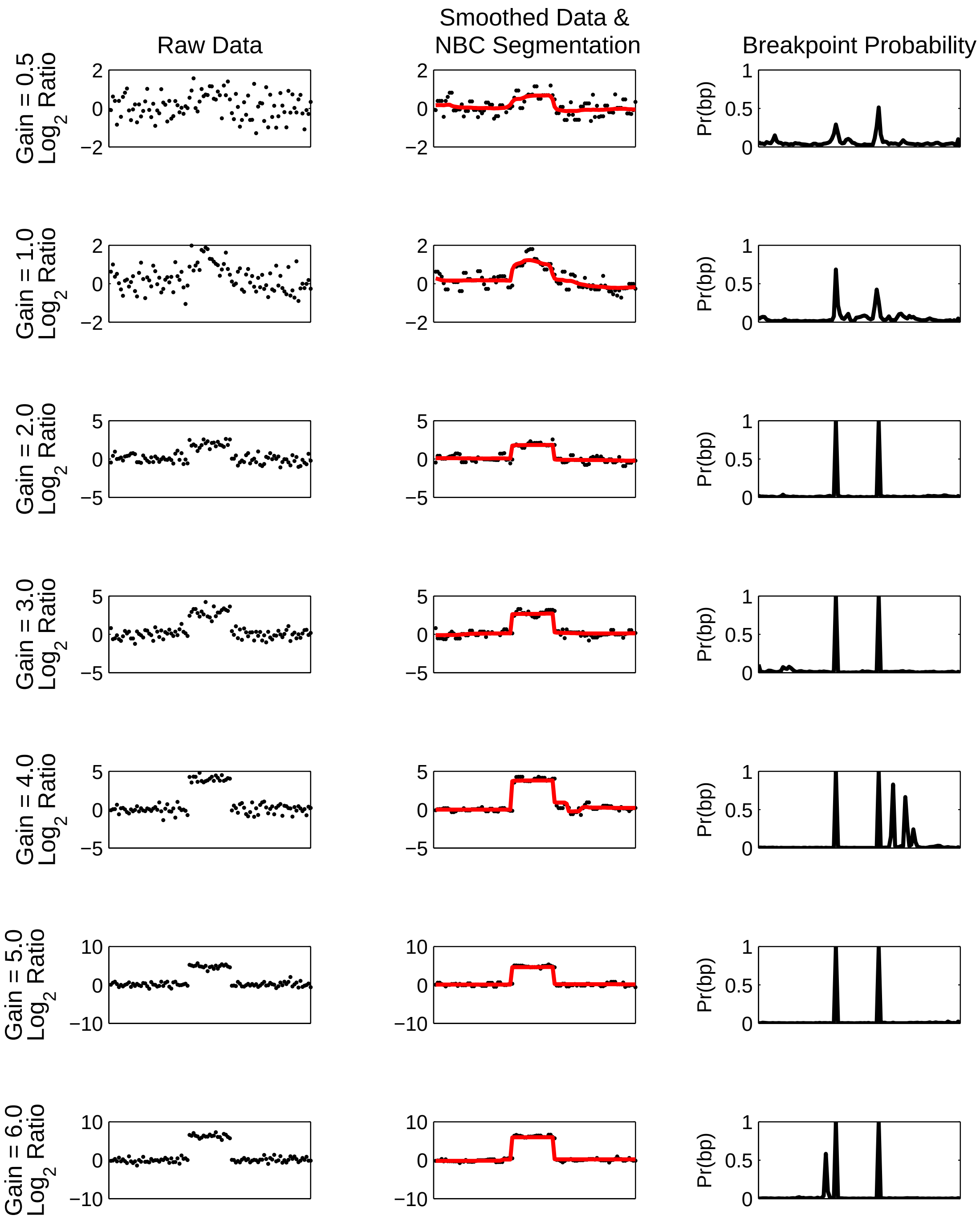

Figure 2: A single artificial chromosome from Simulation #2 with gaussian noise $N(0, 0.5)$ for aberration $\log_2$ ratios of $0.5, 1, 2, 3, 4, 5$ and 6. The first column shows the raw data for a particular aberration $\log_2$ ratio. The second column shows the smoothed data and the average NBC segmentation (red). The third column shows the breakpoint probability at each location.

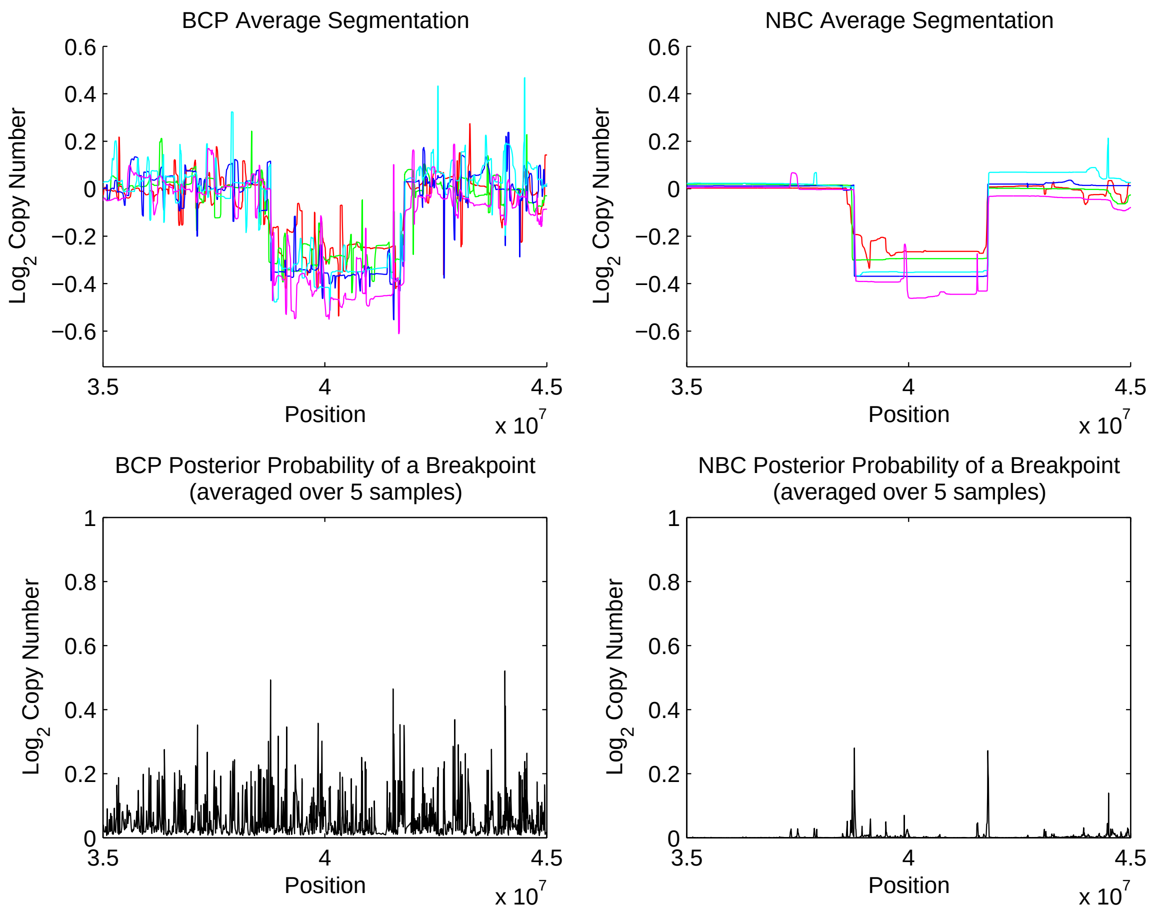

Figure 3: Bayesian segmentations for BCP (Left) compared to NBC (right) for the patients that have the TMPRSS-ERG fusion gene. The mean segmentation is depicted on the top row, and the probability of a breakpoint $P(b_i)$, averaged over the 5 patients, is depicted on the bottom row.

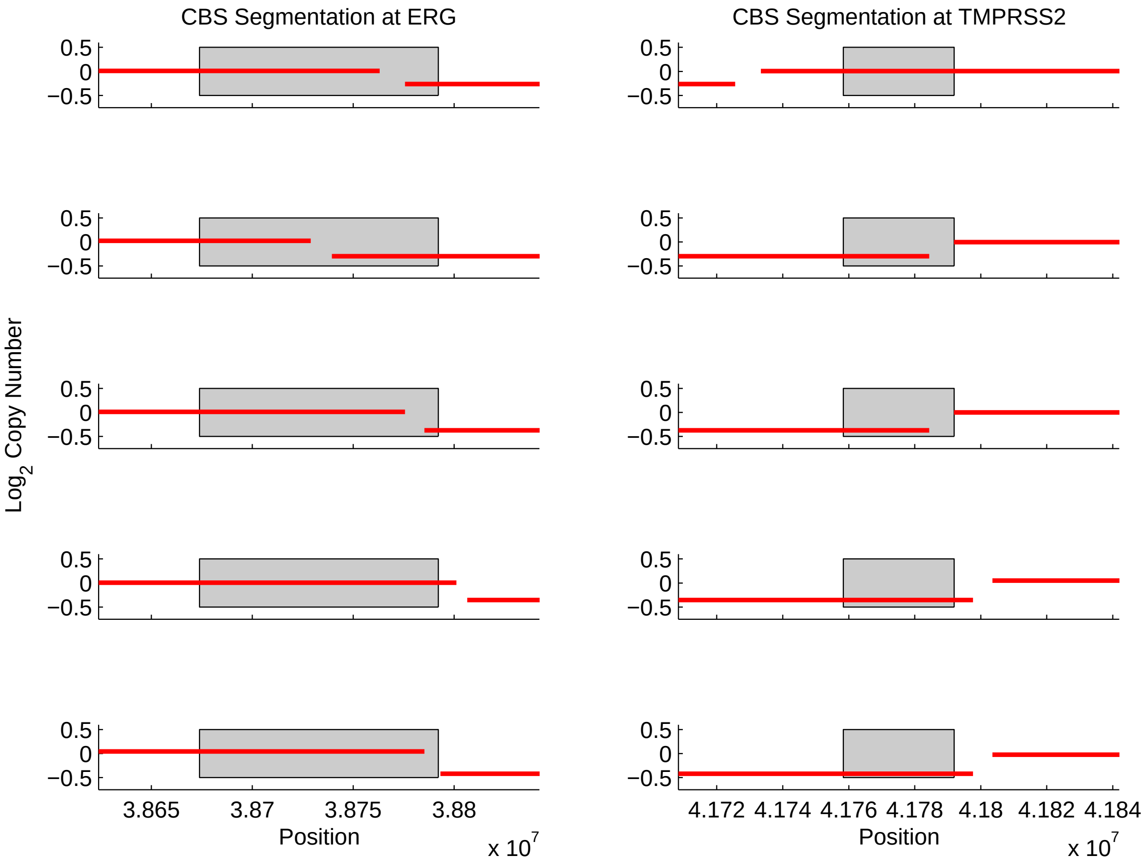

Figure 4: CBS segmentation for the 5 patients that have the TMPRSS-ERG fusion gene according to NBC. Only two of the five individuals have co-occurring breakpoints within the gene regions (gray boxes).

B.2 Comparison to CBS

The CBS implementation in the R package DNAcopy was used to segment the prostate samples. The parameters used were the same as The Cancer Genome Atlas protocol [6]: the data was smoothed using a standard deviation smoothing tecnhique (smooth.CNA) with a smoothing region of 10, 10,000 hybrid permutations were used (nperm-10000,p.method="hybrid"), splits were undone using a standard deviation of 1 (undo.splits="sdundo"" undo.SD=1), and alpha was 0.01. Figure 4 shows the CBS breakpoint locations for the five individuals that NBC reports as having co-occurring breakpoints.

C Implementation Details

We sampled 1,000 breakpoint sequences $\mathbf{A}$ from the posterior distribution of $P(\mathbf{A}|\mathbf{X})$ with $k_{\max} = 100$ possible segments, and set $h_{\min} = 2$ when computing p-values for recurrent breakpoints.

The chromosomes are clearly independent, so we can parallelize the segmentation by considering each chromosome separately. Additionally, we assume that the chromosomal arms are independent as well, implying that there are no real breakpoints at the centromeres. We also assume that there are no real breakpoints in the telomeres. These predictions are ignored.

D Data Access

The Prostate dataset is from UCSF. The Glioblastoma dataset consisted of Level 2 (lowess-normalized) Agilent 244K array data from the Harvard Medical School and was downloaded from the TCGA Data Portal (http://cancergenome.nih.gov/dataportal/ on August 12, 2009. The Prostate dataset uses the hg17 reference, and the GBM dataset uses the hg18 reference. The Database of Genomic Variants was taken from the UCSC Genome Browser (hg17 for Prostate, hg18 for GBM).

References

[1] Daniel Barry and J. A. Hartigan. A bayesian analysis for change point problems. *Journal of the American Statistical Association*, 88(421):309–319, 1993.

[2] Amir Ben-Dor et al. Framework for identifying common aberrations in dna copy number data. *RECOMB 2007*, LNBI(4453):122–136, 2007.

[3] Christopher M. Bishop. *Pattern Recognition and Machine Learning (Information Science and Statistics)*. Springer-Verlag New York, Inc., Secaucus, NJ, USA, 2006.

[4] C. Erdman and J. W. Emerson. A fast Bayesian change point analysis for the segmentation of microarray data. *Bioinformatics*, 24:2143–2148, Oct 2008.

[5] J. S. Liu and C. E. Lawrence. Bayesian inference on biopolymer models. *Bioinformatics*, 15:38–52, Jan 1999.

[6] R. McLendon et al. Comprehensive genomic characterization defines human glioblastoma genes and core pathways. *Nature*, 455:1061–1068, Oct 2008.

[7] R. Walpole, R. Myers, S. Myers, and K. Ye. *Probability & Statistics for Engineers & Scientists*. Prentice-Hall, Upper Saddle River, NJ, 7 edition, 2002.
